# Correction: Systemic and Local Inflammatory Response in Women with Preterm Prelabor Rupture of Membranes

**DOI:** 10.1371/journal.pone.0170862

**Published:** 2017-01-20

**Authors:** Teresa Cobo, Bo Jacobsson, Marian Kacerovsky, David M. Hougaard, Kristin Skogstrand, Eduard Gratacós, Montse Palacio

There are errors in the funding section. The correct funding information is as follows: This work was supported by Instituto de Salud Carlos III (grant number PI10/01308) cofinanced by the Fondo Europeo de Desarrollo Regional de la Unión Europea “Una manera de hacer Europa” (FEDER). The funders had no role in study design, data collection and analysis, decision to publish, or preparation of the manuscript.
