# Correction: Systemic and Local Inflammatory Response in Women with Preterm Prelabor Rupture of Membranes

**DOI:** 10.1371/journal.pone.0095905

**Published:** 2017-10-03

**Authors:** The PLOS ONE Staff

## Notice of Republication

This article was republished on March 27, 2014, due to the figure and tables being published as Supporting Information. Please download the PDF again to view the corrected article. The originally published, uncorrected article and the republished, corrected article are provided here for reference.

## Supporting information

S1 File Originally published, uncorrected article. (PDF)

S2 File Republished, corrected article. (PDF)
