# Supplementary material for: Systemic and Local Inflammatory Response in Women with Preterm Prelabor Rupture of Membranes
Source: PLoS One. 2014 Jan 21;9(1):e85277. doi: 10.1371/journal.pone.0085277 (PMC3897420; doi:10.1371/journal.pone.0085277)
Supplement: Table S1 — Comparison of the levels of proteins (pg/mL) from the maternal serum compartment in women with or without MIAC. (DOC) [file pone.0085277.s001.doc]

**Table S1**. Comparison of the levels of proteins (pg/mL) from the maternal serum compartment in women with or without MIAC

|  | **Maternal serum** | | |
| --- | --- | --- | --- |
|  | **MIAC**  **(n=10)** | **Non-MIAC**  **(n=15)** | ***p*** |
| **IL 8** | 6.5 (4-2635) | 11 (4-675) | 0.495 |
| **IL 18** | 473 (102-775) | 432 (128-2335) | 0.935 |
| **CRP** | 1.73 (0.79-3.47) | 1.5 (0.3-26.1) | 0.338 |
| **RANTES** | 82.4 (40.6-147.9) | 103.5 (35-160) | 0.338 |
| **BDNF** | 8.6 (2.1-10.7) | 8.97 (6.3-29.4) | 0.461 |
| **MMP 9** | 490.6 (394-590) | 447.3 (251.3-552.7) | 0.311 |
| **IFN-γ** | - | - |  |
| **GM-CSF** | - | - |  |
| **sIL-6r** | 23.2 (4.2-71.8) | 23.2 (12.9-72.3) | 0.978 |
| **Mip 1α** | 10 (10-1274) | 29 (10-122) | 0.338 |
| **IL 6** | 22.5 (10-752) | 20 (4-277) | 0.849 |
| **IL 10** | 79 (4-232) | 77 (4-203) | 0.849 |
| **IL 12** | 11.5 (4-33) | 15 (9-25) | 0.261 |
| **IL 1β** | 25 (4-78) | 36 (13-91) | 0.238 |
| **TNF β** | 711.5 (213-1725) | 915 (376-1915) | 0.567 |
| **MCP 1** | 105.5 (10-280) | 73 (4-160) | 0.160 |
| **TREM 1** | 488 (488-5970) | 488 (488-4859) | 0.367 |
| **NT3** | 57.5 (4-254) | 49 (4-469) | 0.216 |
| **Adinopectin** | 15.8 (5.7-35.6) | 11.3 (8.3-35.3) | 0.643 |
| **IGFBP1** | 78.2 (12.1-100) | 42.3 (7.5-100) | 0.216 |
| **IGFBP3** | 709.5(283.3-1894.4) | 841.9(318.1-1206.1) | 0.338 |
| **Leptin** | 26.1 (3.1-100) | 43.8 (12.3-100) | 0.261 |
| **sTNF R1** | 1.19 (0.48-6.24) | 1.6 (0.82-3.85) | 0.238 |
| **MIF** | 5 (0.09-27.4) | 7.3 (0.09-20.4) | 0.849 |

MIAC: microbial invasion of the amniotic cavity. Continuous variables: Mann-Whitney *U* test, median (range).
