# Supplementary material for: Correction: Systemic and Local Inflammatory Response in Women with Preterm Prelabor Rupture of Membranes
Source: PLoS One. 2017 Oct 3;12(10):e0095905. doi: 10.1371/journal.pone.0095905 (PMC5626023; doi:10.1371/journal.pone.0095905)
Supplement: S1 File — (PDF) [file pone.0095905.s001.pdf]

PLOS ONE

Systemic and Local Inflammatory Response in Women with Preterm Prelabor Rupture of Membranes

Teresa Cobo[1]*, Bo Jacobsson[2,3], Marian Kacerovsky[4], David M. Hougaard[5], Kristin Skogstrand[5], Eduard Gratacós[1], Montse Palacio[1]

1 Maternal-Fetal Medicine Department, ICGON, Hospital Clınic, Universitat de Barcelona, Barcelona, Spain; Fetal and Perinatal Medicine Research Group, Institut d'Investigacions Biomediques August Pi i Sunyer (IDIBAPS), Barcelona, Spain; Centro de Investigación Biomédica en Red de Enfermedades Raras (CIBERER), Barcelona, Spain, **2** Department of Obstetrics and Gynecology, Sahlgrenska University Hospital, Gothenburg, Sweden, **3** Institute of Public Health, Oslo, Norway, **4** Department of Obstetrics and Gynecology, University Hospital Hradec Kralove, Czech Republic, **5** Department of Clinical Biochemistry and Immunology, Statens Serum Institut, Copenhagen, Denmark

Abstract

Objective: To evaluate the inflammatory pattern in maternal circulation, amniotic cavity, cervix and vagina from women with preterm prelabor rupture of membranes (PPROM) considering the occurrence of microbial invasion of the amniotic cavity (MIAC).

Methodology: A prospective study was performed in 58 women with PPROM before 34+0 weeks of gestational age. Twenty-six proteins were analyzed by a multiple immunoassay in samples of amniotic fluid, serum, cervix and vagina. Association of an inflammatory response in the invasive and non-invasive samples with MIAC was investigated.

Results: The rate of MIAC was 36.2% (21/58). Both amniotic fluid IL-6 and cervical C-reactive protein (CRP) showed to be independent predictors of MIAC. A cut-off level of cervical CRP≥1836 pg/mL showed a detection rate of 75%, false positive rate of 19% and positive and negative predictive values to predict MIAC of 67% and 87%, respectively. There were no independent biomarkers of MIAC either in the serum or vaginal compartment.

Conclusion: A cervical inflammatory response mediated by CRP was observed in PPROM women with MIAC. Evaluation of serum or vaginal samples did not add valuable information regarding the outcome evaluated.

Citation: Cobo T, Jacobsson B, Kacerovsky M, Hougaard DM, Skogstrand K, et al. (2014) Systemic and Local Inflammatory Response in Women with Preterm Prelabor Rupture of Membranes. PLoS ONE 9(1): e85277. doi:10.1371/journal.pone.0085277

Editor: Roger C. Young, University of Tennessee Health Science Center, United States of America

Received July 9, 2013; **Accepted** November 25, 2013; **Published** January 21, 2014

Copyright: © 2014 Cobo et al. This is an open-access article distributed under the terms of the Creative Commons Attribution License, which permits unrestricted use, distribution, and reproduction in any medium, provided the original author and source are credited.

Funding: This work was supported by a Rio Hortega grant from Instituto de Salud Carlos III in Spain (CM09/00213) and grants from Spanish government (FIS PI10/01308). The funders had no role in study design, data collection and analysis, decision to publish, or preparation of the manuscript.

Competing Interests: The authors have declared that no competing interests exist.

* E-mail: tcobo@clinic.ub.es

Introduction

Preterm prelabor rupture of membranes (PPROM) is responsible for approximately one-third of spontaneous preterm deliveries and for a substantial proportion of neonatal morbidity and mortality in relation to gestational age and the risk of infection [1].

Several inflammatory biomarkers alone or in combination have been previously reported as predictors of microbial invasion of the amniotic cavity (MIAC) in the amniotic fluid compartment in women with preterm labor either with intact membranes (PTL) or with PPROM [2–9]. However, there are few references about the role of these inflammatory biomarkers in non-invasive samples, such as serum [5,10], cervix [11–13] or vagina [14], with most being focused on women with PTL.

Remarkably, few studies have been carried out in the subgroup of women with PPROM with poor predictive capacity to predict MIAC by non-invasive fluids [10,12]. Few information is available on this topic and studies are needed to determine whether the inflammatory status in non-invasive samples predict either the inflammatory/infectious status in the amniotic cavity. Furthermore, the identification of non-invasive inflammatory biomarkers in women with PPROM is relevant from a clinical point of view, considering not only the risk but the difficulty of an invasive procedure to obtain amniotic fluid in these women, most of them complicated with oligohydramnios.

Therefore, the aim of this study was to investigate the pattern of inflammation not only in the amniotic fluid compartment but most interesting in non-invasive compartments as serum, cervix and vagina in women with PPROM using a panel of inflammatory proteins determined by a multiple immunoassay and to evaluate its relationship with MIAC.

Material and Methods

Study population

From September 2005 to June 2008, women with a diagnosis of PPROM before 34.0 weeks admitted to the Maternal-Fetal Medicine Department at the Hospital Clinic in Barcelona were considered eligible for the study. PPROM was defined as leakage of amniotic fluid preceding the onset of uterine contractions. PPROM was diagnosed by a sterile speculum examination to

identify pooling of amniotic fluid in the vagina and was confirmed by alkaline pH in the absence of vaginal infection. Gestational age was calculated based on the crown-rump length at first trimester ultrasound. Multiple pregnancies, structural/chromosomal anomalies, clinical signs of chorioamnionitis [15], vaginal bleeding at admission or those cases where amniocentesis was not possible (i.e.anhydramnios) were excluded.

According to our standard protocol, blood sampling and a transabdominal amniocentesis to rule out signs of chorioamnionitis, cervical cultures for the identification of bacteria and fungi, and transvaginal cervical length by ultrasound, were performed at admission. The amniocentesis was performed before the administration of corticosteroids or antibiotics.

Amniotic fluid cultures for aerobic, anaerobic bacteria and genital mycoplasma, as well as glucose concentration were performed immediately after collection and their results were available for clinical management. A complete course of antenatal steroids, betamethasone 12 mg intramuscular injection with two doses given 24 h apart, was administered when PPROM occurred from 24.0 to 34.0 weeks. Tocolysis was only considered if uterine contractions in the absence of clinical chorioamnionitis, abruption placentae and fetal compromise. Prophylactic parenteral broad-spectrum antibiotics were given for a period of 5 days. The antibiotic regimen of choice in our institution at that time was parenteral ampicillin 1 g every 6 h and gentamycin 80 mg every 8 h. In line with other clinical management guidelines1, labor was induced if PPROM occurred at or beyond 34.0 weeks. If PPROM occurred before 31.0 weeks, expectant management was performed. According to our local protocol at that time, at or beyond 31.0 weeks of gestation, labor was induced if pulmonary maturity was documented.

Women with a positive cervical culture were correctly treated orally or vaginally according to the microorganism isolated. MIAC was defined as a positive amniotic fluid culture. Women with MIAC were treated with parenteral antibiotics during 10 days according to the antibiogram of the microorganism isolated. Maternal and fetal status were closely monitored for signs of chorioamnionitis, labor and/or fetal compromise. Induction of labor was indicated based on virulence of the microorganism, gestational age, maternal or fetal clinical signs of chorioamnionitis. Spontaneous preterm delivery was defined as delivery following spontaneous labor.

To identify inflammatory biomarkers in non-invasive samples from the cervical and vaginal compartment, cervical and vagina mucus were obtained separately using Cytobrush swabs (Cytobrush Plus GT; Medscan Medical AB) from the external cervical os and from the 6 middle third of the vagina, respectively after amniocentesis sampling. Each Cytobrush was submerged in 1.0 ml of NaCl (9 mg/ml) and kept at 4°C until processing, which occurred within 2 hours. Cervical, vaginal and the remaining amniotic fluid and serum samples were centrifuged at 4000 rpm at 4°C for 10 minutes and stored at −80°C until analysis.

Twenty-six proteins were simultaneously analyzed by a multiple immunoassay in amniotic fluid, maternal serum, cervix and vagina. Details about the methodology used for proteomic biomarkers determinations and threshold used were available in a supplemental appendix. An interim analysis was planned with the first 25 serum and amniotic fluid samples. Main variable outcome was MIAC.

Ethics statement

Written informed consent was obtained from all subjects. The Institutional Review Board approved the collection and use of these samples for research purposes (Ethics Committee of Hospital Clinic of Barcelona 2005/56).

Statistical methods

Statistical analyses were performed using SPSS 19.0 for Windows XP OS (SPSS Inc., USA). Demographic and clinical characteristics were compared using the nonparametric Mann-Whitney U test and the results are presented as median (range). Categorical variables were compared using linear by linear association Chi-square and are presented as numbers (%). All significant continuous variables were entered into a logistic regression with a forward selection. Receiver operator curves were constructed to determine the best cut-off value to predict the outcome. Differences were considered statistically significant with a $p<0.05$ with two-sided alternative hypotheses.

Results

Over the study period, 58 women met the inclusion criteria and accepted to be included in the study. Distribution according the occurrence of MIAC in the different non-invasive compartments is shown in Table S1.

The maternal and neonatal characteristics are summarized in Table S2. Gestational age at PPROM was less than 24.0 weeks in 8 (14%) women; between 24.0 and 27.6 weeks in 16 (27%) women; between 28.0 and 31.6 weeks in 18 (31%) women and beyond 32.0 weeks in 16 (27%) women. Gestational age at sampling and amniotic fluid glucose was significantly lower in women with MIAC than without. These two significant variables were entered in a logistic regression with a forward selection with the significant invasive and non-invasive inflammatory proteins to assess the independent biomarker of MIAC.

The overall rate of MIAC was 36.2% (21/58). The most common microorganism isolated from amniotic fluid was *Ureaplasma urealyticum* (n = 10). Other microorganisms isolated were *Mycoplasma hominis* (n = 1), *Streptococcus agalactiae* (n = 1), *Streptococcus viridans* (n = 3), *Streptococcus pneumoniae and Haemophylus influenzae* (n = 1), *Escherichia coli* (n = 1), *Candida albicans* (n = 1), *Fusobacterium* (n = 1), *Gardnerella vaginalis* (n = 1) and *Candida glabrata* (n = 1).

Three out of 21 women with MIAC presented a positive cervical culture at admission: *Candida albicans* and *Escherichia coli* (n = 1), *Candida glabrata* (n = 1), and *Gardnerella vaginalis* (n = 1). Six out of 37 women without MIAC presented a positive cervical culture by *Candida albicans and Escherichia coli* (n = 3) and *Gardnerella vaginalis* (n = 3).

From 26 biomarkers evaluated, maternal serum interferon-(IFN)-γ and granulocyte macrophage colony stimulating factor (GM-CSF) and amniotic fluid, cervical and vaginal Interleukin (IL)-17 and tumor necrosis factor (TNF)-α had undetectable levels in more than 50% of the samples and were subsequently excluded from further analyses.

The univariate analysis of the serum samples in the first 25 women revealed that there were no significant differences in none of the inflammatory proteins of interest in women with MIAC (Table S3). Therefore, remaining maternal blood samples were not analyzed. From then onwards, only samples simultaneously obtained from the amniotic fluid, the cervix and the vagina were considered for analysis. Amniotic fluid levels of IL-8, C-reactive protein (CRP), regulated on activation normal T-expressed and secreted (RANTES), brain-derived neurotropic factor (BDNF), IFN-γ, GM-CSF, macrophage inflammatory protein-1α (MIP-1α), IL-6, IL-10, IL-1β, TNF-β, triggering receptor expressed on myeloid cells-1 (TREM-1), neutropin-3 (NT3), and soluble TNF receptor-1 (sTNF-R1) were significantly higher in women with

MIAC than without (Table S4). However, when we considered other significant clinical variables (gestational age at sampling and amniotic fluid glucose) only amniotic fluid IL-6 (OR 95% Confidential interval (CI): 1.002 (1.000–1.003); p 0.019) remained independent predictor of MIAC.

Cervical levels CRP, RANTES, IL-1β, TREM-1 and NT3 were significantly higher in women with MIAC than without but only cervical CRP (OR 95% CI: 1.001 (1.000–1.002); p 0.04) showed to be an independent biomarker in the multivariate analysis. Receiver operator curve (ROC) analysis demonstrated that the best cut-off value for cervical CRP to predict MIAC was 1836 pg/mL (area under the curve 0.77, 95% Confidence Interval: 0.59–0.89) with a sensitivity of 75%, specificity of 81%, positive predictive value of 67%, negative predictive value of 87% and positive and negative likelihood ratio of 4.0 and −0.307, respectively.

Finally, although vaginal CRP levels were significantly higher in women with MIAC than without, only gestational age at sampling remained significantly independent (OR 0.680, 95% CI (0.480–0.962); p 0.29) when considered all significant variables in the multivariate analysis.

Discussion

The main finding of the study was the existence of an inflammatory response in the cervical compartment mediated by CRP in PPROM women with MIAC but not in the serum and vaginal compartment.

In line with previously published in preterm labor with or without membranes rupture [2,3,5–8,16,17], a significant inflammatory response in the amniotic fluid was observed in response to MIAC, being IL-6 its best predictor.

Regarding non-invasive compartments, a different inflammatory pattern was observed only in the cervical compartment in women with MIAC compared to women without. In line with previously reported with cervical IL-6 [12], the presence of a high concentration of CRP in the cervical compartment accurately reflected the presence of an inflammatory/infectious status in the amniotic cavity with a high detection rate to predict MIAC of 75%, a relatively low FP rate (13%) and a high PPV (75%). These findings are clinically relevant due to the feasibility and accessibility of the assessment of cervical mucus in clinical practice and support the notion that a single test would be useful to provide rapid prediction of MIAC in patients with PPROM without clinical symptoms of chorioamnionitis.

On the other hand, we did not find a different expression of inflammatory proteins in response to MIAC either in the vaginal or in the serum compartment, suggesting that these non-invasive compartments do not seem to translate the inflammatory status of the amniotic cavity. This is in contrast to the findings of Hitti et al. [14] who in a subgroup of women with PTL with intact membranes, identified proteins in the vaginal fluid compartment capable of discriminating between women with and without MIAC. Differences between the two populations explored (PTL vs. PPROM) and different proteomic biomarkers analysed could partially explain why differences in the proteomic profile identified in vagina by Hitti et al. [14], were not found in our study. In the serum compartment, preliminary data reported by Gravett et al. [5] suggested that proteomic biomarkers such as calgranulin B and IGFBP-1 might be detectable in maternal serum. When 26 inflammatory proteins were simultaneously evaluated using multiple immunoassay in a cohort of PPROM women with MIAC, a low systemic inflammatory response was observed by our group, mediated only by maternal serum IL-18, IL-1β and MCP-1 [10]. Despite a similar approach was performed in the current study, we did not find any significant differences in the serum compartment.

The main strength of the study was the assessment of biological samples (maternal serum, cervix and vagina) obtained simultaneously to amniotic fluid sampling. This may provide better knowledge of the inflammatory response to infection in the different compartments at the same time. Furthermore, to our knowledge, there are no previous studies evaluating the inflammatory process using such a broad panel of biomarkers in non-invasive samples in PPROM women.

Our study also presents some limitations. First, it was performed in a single institution thereby limiting the sample size. Secondly, although amniotic fluid was collected in all women, not all non-invasive samples (serum, cervix and vagina) were assessed in all of them limiting again the sample size. And finally, short and long term neonatal outcomes were not considered and therefore, the impact on neonatal outcomes regarding the presence or absence of an inflammatory status is not known.

Conclusion

In summary, cervical inflammation mediated by CRP seems to be a good predictor of MIAC while samples from serum and vagina seem to add no value. Since there is a limitation with sample size, further studies must be considered to ratify these preliminary results.

Supporting Information

Table S1 Clinical distribution of the study population.
(DOC)

Table S2 Maternal characteristics according to MIAC in women with PPROM.
(DOC)

Table S3 Comparison of the levels of proteins (pg/mL) from the maternal serum compartment in women with or without MIAC.
(DOC)

Table S4 Comparison of the levels of proteins (pg/mL) in PPROM women with and without MIAC.
(DOC)

Author Contributions

Conceived and designed the experiments: TC MP BJ MK EG. Performed the experiments: KS DH. Analyzed the data: TC MP. Contributed reagents/materials/analysis tools: TC MP BJ MK. Wrote the paper: TC.

References

1. ACOG (2007) Practice Bulletin No. 80: premature rupture of membranes. Clinical management guidelines for obstetrician-gynecologists. Obstet Gynecol Apr;109(4):1007–19.
2. Buhimschi CS, Bhandari V, Hamar BD, Bahtiyar MO, Zhao G, et al.(2007) Proteomic profiling of the amniotic fluid to detect inflammation, infection, and neonatal sepsis. PLoS Med Jan;4(1):e18.
3. Buhimschi CS, Buhimschi IA, Abdel-Razeq S, Rosenberg VA, Thung SF, et al. (2007) Proteomic biomarkers of intra-amniotic inflammation: relationship with funisitis and early-onset sepsis in the premature neonate. Pediatr Res Mar;61(3):318–24.
4. Buhimschi IA, Christner R, Buhimschi CS. (2005) Proteomic biomarker analysis of amniotic fluid for identification of intra-amniotic inflammation. Bjog Feb;112(2):173–81.

5. Gravett MG, Novy MJ, Rosenfeld RG, Reddy AP, Jacob T, et al. (2004) Diagnosis of intra-amniotic infection by proteomic profiling and identification of novel biomarkers. Jama Jul 28;292(4):462–9.
6. Jacobsson B, Mattsby-Baltzer I, Andersch B, Bokstrom H, Holst RM, et al. (2003) Microbial invasion and cytokine response in amniotic fluid in a Swedish population of women with preterm prelabor rupture of membranes. Acta Obstet Gynecol Scand May;82(5):423–31.
7. Jacobsson B, Mattsby-Baltzer I, Andersch B, Bokstrom H, Holst RM, et al. (2003) Microbial invasion and cytokine response in amniotic fluid in a Swedish population of women in preterm labor. Acta Obstet Gynecol Scand Feb;82(2):120–8.
8. Cobo T, Palacio M, Navarro-Sastre A, Ribes A, Bosch J, et al. (2009) Predictive value of combined amniotic fluid proteomic biomarkers and interleukin-6 in preterm labor with intact membranes. Am J Obstet Gynecol. May;200(5):499 e1–6.
9. Cobo T, Palacio M, Martinez-Terron M, Navarro-Sastre A, Bosch J, et al. (2011) Clinical and inflammatory markers in amniotic fluid as predictors of adverse outcomes in preterm premature rupture of membranes. Am J Obstet Gynecol Aug;205(2):126e1–8 Epub 2011 Apr 8 2011 Apr 8.
10. Cobo T, Tsiartas P, Kacerovsky M, Holst RM, Hougaard DM, et al. (2012) Maternal inflammatory response to microbial invasion of the amniotic cavity: analyses of multiple proteins in the maternal serum. Acta Obstet Gynecol Scand Jan;92(1):61–8.
11. Holst RM, Hagberg H, Wennerholm UB, Skogstrand K, Thorsen P, et al. (2011) Prediction of microbial invasion of the amniotic cavity in women with preterm labour: analysis of multiple proteins in amniotic and cervical fluids. BJOG Jan;118(2):240–9.
12. Jun JK, Yoon BH, Romero R, Kim M, Moon JB, et al. (2000) Interleukin 6 determinations in cervical fluid have diagnostic and prognostic value in preterm premature rupture of membranes. Am J Obstet Gynecol Oct;183(4):868–73.
13. Rizzo G, Capponi A, Rinaldo D, Tedeschi D, Arduini D, et al. (1996) Interleukin-6 concentrations in cervical secretions identify microbial invasion of the amniotic cavity in patients with preterm labor and intact membranes. Am J Obstet Gynecol Oct;175(4 Pt 1):812–7.
14. Hitti J, Lapidus JA, Lu X, Reddy AP, Jacob T, et al. (2010) Noninvasive diagnosis of intraamniotic infection: proteomic biomarkers in vaginal fluid. Am J Obstet Gynecol Jul;203(1):32 e1–8. 20.
15. Gibbs RS, Blanco JD, St Clair PJ, Castaneda YS. (1982) Quantitative bacteriology of amniotic fluid from women with clinical intraamniotic infection at term. J Infect Dis Jan;145(1):1–8.
16. Shim SS, Romero R, Hong JS, Park CW, Jun JK, et al. (2004) Clinical significance of intra-amniotic inflammation in patients with preterm premature rupture of membranes. Am J Obstet Gynecol Oct;191(4):1339–45.
17. Yoon BH, Romero R, Moon JB, Shim SS, Kim M, et al. (2001) Clinical significance of intra-amniotic inflammation in patients with preterm labor and intact membranes. Am J Obstet Gynecol Nov;185(5):1130–6
